# DNA damage-induced translocation of S100A11 into the nucleus regulates cell proliferation

**DOI:** 10.1186/1471-2121-11-100

**Published:** 2010-12-17

**Authors:** Theresa Gorsler, Ulrike Murzik, Tobias Ulbricht, Julia Hentschel, Peter Hemmerich, Christian Melle

**Affiliations:** 1 Core Unit Chip Application (CUCA), Institute of Human Genetics and Anthropology, University Hospital Jena, 07740 Jena, Germany; 2 Department of Molecular Biology, Fritz Lipmann Institut (FLI) - Leibniz Institute for Age Research, 07743 Jena, Germany; 3 Current Address: Abt. Molekulare Onkologie, Universitätsmedizin Göttingen, Georg-August-Universität, 37077 Göttingen, Germany; 4 Current Address: Membrane Trafficking Group; Fritz Lipmann Institut (FLI) - Leibniz Institute for Age Research, 07743 Jena, Germany; 5 Current Address: Biomolecular Photonics Group, University Hospital Jena, 07740 Jena, Germany

## Abstract

**Background:**

Proteins are able to react in response to distinct stress stimuli by alteration of their subcellular distribution. The stress-responsive protein S100A11 belongs to the family of multifunctional S100 proteins which have been implicated in several key biological processes. Previously, we have shown that S100A11 is directly involved in DNA repair processes at damaged chromatin in the nucleus. To gain further insight into the underlying mechanism subcellular trafficking of S100A11 in response to DNA damage was analyzed.

**Results:**

We show that DNA damage induces a nucleolin-mediated translocation of S100A11 from the cytoplasm into the nucleus. This translocation is impeded by inhibition of the phosphorylation activity of PKCα. Translocation of S100A11 into the nucleus correlates with an increased cellular p21 protein level. Depletion of nucleolin by siRNA severely impairs translocation of S100A11 into the nucleus resulting in a decreased p21 protein level. Additionally, cells lacking nucleolin showed a reduced colony forming capacity.

**Conclusions:**

These observations suggest that regulation of the subcellular distribution of S100A11 plays an important role in the DNA damage response and p21-mediated cell cycle control.

## Background

Cells are exposed to changing environmental conditions that can cause cellular stress. Stress-inducing situations include severe variations of the cellular energy budget, altered concentration of specific ions and also conditions that induce DNA damage. In case of DNA damage, cell cycle arrest or illegitimate DNA rearrangements, cell death or carcinogenesis can occur if cellular systems fail to repair the DNA properly [1]. As a consequence, the integrity of the genome is threatened. Response mechanisms of cells to genotoxic stress include directed intracellular trafficking of specific proteins mediated commonly by posttranslational modifications as well as formation of specific protein-protein interactions [2-4]. In a recent study, we showed a functional cooperation of S100A11 with the repair machinery at sites of DNA double-strand breaks (DSBs) [5]. S100A11 belongs to the family of S100 proteins which are considered as multitasking proteins involved in several biological processes such as the Ca^2+ ^signalling network, cell growth and motility, cell cycle progression, transcription and cell differentiation [6-8]. It has been proposed that the S100 proteins are involved in the differentiation of specific tissues and that some members of this family are differentially expressed in normal human skin and melanocytic lesions [9]. S100 proteins are expressed in a cell and tissue specific manner [10]. In several studies, S100A11 was shown to be up- or down-regulated in different tumor entities [11,12]. S100A11 plays a dual role in growth regulation of human keratinocytes as it is able to mediate a Ca^2+^-induced growth inhibition as well as growth stimulation by enhancement of the level of EGF protein family members [13,14]. Interestingly, the stimulation of the activity of the cell cycle regulator p21^WAF1/CIP1 ^by potential cellular stress stimuli such as increase of extracellular Ca^2+ ^concentration as well as induction of DNA damage can be mediated by S100A11 through a p53 independent mechanism [5,13].

The aim of the present study was to gain further mechanistic insight into the role of S100A11 cellular trafficking during the DNA damage response pathway.

## Methods

### Cell culture

The human keratinocyte cell line HaCaT [15] and human U-2 OS osteosarcoma cells were cultured in DMEM supplemented with 10% fetal bovine serum. Cells were grown to 80% confluence and passaged at a split ratio of 1:4. For western blot experiments, cells were harvested at 70-90% confluency and lysed in a buffer containing 100 mM sodium phosphate pH 7.5, 5 mM EDTA, 2 mM MgCl_2_, 0.1% CHAPS, 500 μM leupeptin, and 0.1 mM PMSF. After centrifugation (15 min; 15000 rpm) the supernatant was immediately applied to SDS-PAGE. Preparations of cytoplasmic and nuclear cell fractions were performed using the ProtoJET cytoplasmic and nuclear protein extraction kit (Fermentas) according to the manufactor's instructions.

### Construction of the GFP-S100A11 plasmid

An S100A11 construct from a pGEX-2T-S100A11 vector (kindly provided by Dr. N.H. Huh, Okayama University) was PCR amplified using following primers: 5'-gcttcgaattctatggcaaaaatctccagccc-3' (sense) and 5'-ggtggatccggtccgcttctgggaaggga-3' (antisense). The PCR fragment was cloned between the EcoR1 and BamH1 restriction site of pEGFP-C1 (Clontech). Correct insertion of S100A11 was confirmed by sequencing.

### siRNA mediated knockdown of nucleolin

Small interfering RNA (siRNA) duplex oligonucleotides used in this study are based on the human cDNAs encoding nucleolin. Nucleolin siRNA as well as a non-silencing control siRNA were obtained from QIAGEN GmbH (Hilden, Germany). The siRNA sequence applied to target nucleolin was 5'-AAG AAC GTG GCT GAG GAT GAA-3'. The siRNA sequences employed as negative controls were 5'-UUC UCC GAA CGU GUC ACG UdTdT-3' (sense) and 5'-ACG UGA CAC GUU CGG AGA AdTdT-3' (antisense). HaCaT cells (2 × 10^5^) were plated on 6-well plates 18 hours prior to transfection and were 50% confluent when siRNA was added. The amount of siRNA duplexes applied was 1.5 μg/well for nucleolin. Transfection was performed using the amphiphilic delivery system SAINT-RED (Synvolux Therapeutics B.V., Groningen, The Netherlands) as described [5]. Briefly, siRNA was complexed with 15 nmol of transfection reagent and added to the cells for 4 hours. Subsequently, 2 ml of culture medium was added and incubation proceeded for 72 hours.

### Antibodies for immunofluorescence-based microscopy

Anti-S100A11 chicken polyclonal antibody (ab15612; Abcam), anti-nucleolin mouse monoclonal antibody (4E2; MBL) and anti-γH2AX rabbit polyclonal antibody raised in rabbits against a phosphorylated peptide corresponding to the C-terminus of human γH2AX were used in two- or three-color immunofluorescence staining as primary antibodies which were detected with species-specific secondary antibodies linked to fluorescein, Cy3 or Cy5 (Dianova).

### Immunocytochemistry and confocal microscopy

Cells grown on coverslips were fixed by treatment with methanol at -20°C for 5 min followed by acetone (prechilled to -20°C) for 3 min, or by incubation in 4% paraformaldehyde for 10 min at room temperature followed by 25% Triton-X 100 for 3 min. Immunofluorescence was performed as previously described [5]. Samples were scanned with a Zeiss LSM 510 laser scanning confocal device attached to an Axioplan 2 microscope using a 63× Plan-Apochromat oil objective (Carl Zeiss, Jena, Germany). Fluorescein, Cy3 or Cy5 dyes were excited by laser light at a 488-, 552-, or 633-nm wavelength, respectively. To avoid bleed-through effects in double or triple staining experiments, each dye was scanned independently using the multitracking function of the LSM 510 unit. Single optical sections were selected either by eye-scanning the sample in z axis for optimal fluorescence signals, or selected from z-stacks. Images were electronically merged using the LSM 510 (Carl Zeiss, Jena, Germany) software and stored as TIFF files. Figures were assembled from the TIFF files using Adobe Photoshop software.

### Induction of DNA damages by bleomycin (BLM)

Cells were seeded in 6-well plates on cover slips for 16 hours. DMEM supplemented with 10% FCS was exchanged to fresh DMEM supplemented with 10% FCS and cells were treated with 12.5 IU/ml BLM. Medium was exchanged after 30 minutes and cells were analyzed after different time points. DNA double-strand breaks (DSBs) were visualized by immunofluorescence using a specific antibody against γH2AX. H2AX becomes phosphorylated as one of the first cellular responses after DNA damage and forms foci at sites of DSBs [16].

### Quantification of S100A11 translocation

The GFP-S100A11 fluorescence signal was quantified from maximum intensity projections of 3-D image stacks of U-2 OS cells treated with BLM for 30 min or in untreated control cells, Quantification was done with MetaMorph analysis software (MDS Analytical Technologies). Fluorescence intensity of GFP-S100A11 in five cytoplasmic areas (2 μm diameter each) localized along a straight line from the nuclear membrane to the cellular periphery in four different directions based on the nuclear membrane as well as of three areas randomly selected in the nucleus was measured.

### Western blot

Proteins of interest in crude extracts of U-2 OS and HaCaT cells treated with specific nucleolin siRNA or unspecific control siRNA were verified using specific antibodies against nucleolin (mouse monoclonal, 4E2; MBL), actin (rabbit polyclonal, A266; Sigma), S100A11 (rabbit polyclonal,10237-1-AP; Protein Tech Group), Ku70 (mouse monoclonal, AB-4/N3H10; Labvision), tubulin (rabbit polyclonal, ab18251; Abcam), and p21 (rabbit polyclonal, sc-469; Santa Cruz) by Western blot assays as described.

### qRT-PCR

HaCaT cells transfected with specific nucleolin or control siRNA were treated with BLM (12.5 IU/μl) for 1 h. Total RNA was extracted using an RNA Isolation Kit (Qiagen, Germany) and first strand synthesis was synthesized using a Kit system (Fermentas, Germany) according to the manufacturer's instructions. The mRNA level of the p21 gene was estimated by quantitative real-time PCR using specific primers: p21 fwd 5' CTG TCA CTG TCT TGT ACC CTT GT 3'; p21 rev 5' CTT CCT GTG GGC GGA TTA G 3'. The CT value of the p21 gene was normalized to actin.

### Colony forming assay

To assess the survival rate of cells after bleomycin treatment, 10^4 ^HaCaT cells were seeded into 6-well dishes. After 24 hours, the cells were treated with different concentrations of the drug and cultured for another ten days. In control treatments, single cells had formed colonies of about 30 cells after that time. Colonies were than washed once with PBS, fixed with methanol for 15 min, stained with Giemsa dye, and finally air-dried. The number of colonies formed was then determined.

## Results

### Nuclear translocation of S100A11 is induced by stress stimuli

**Figure 1:**
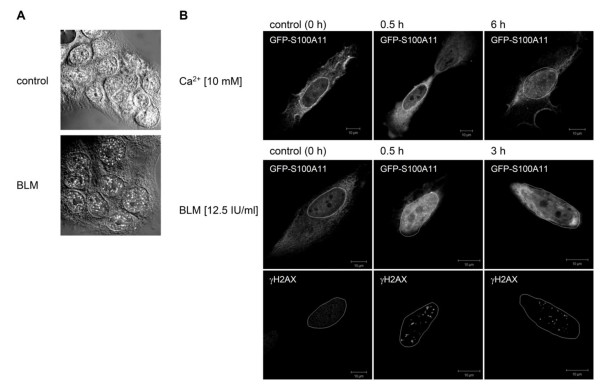
**Translocation of S100A11 into the nucleus of human U-2 OS cells after stress stimulation**. (A) Fixed human HaCaT keratinocytes treated with bleomycin (BLM) for 30 min were immunostained with anti-S100A11 antibody. In BLM treated cells decreased staining in the cytoplasm as well as increased staining in the nucelus can be observed. (B) U-2 OS cells transfected with GFP-S100A11 were treated with CaCl_2 _(10 mM) or BLM (12.5 IU/ml), and analyzed by immunostaining followed by laser scanning microscopy for GFP-S100A11 and for γH2AX at different time points at indicated.

It was shown recently that S100A11 is involved in the detection of DNA double-strand breaks (DSBs) by the DNA repair machinery [5]. When human HaCaT keratinocytes were treated with bleomycin (BLM), a DNA double-strand break (DSB)-inducing agent, an increased accumulation of S100A11 at discrete foci in the nucleus, accompanied by a decreased staining in the cytoplasm was detectable compared to untreated control cells (Figure 1A). Consistent with previous observations [5], we detected a distribution of endogenous S100A11 in a dot-like pattern in the nucleus of DNA damaged cells. This behaviour of S100A11 is reminiscent of its nuclear translocation induced by high Ca^2+ ^as reported previously [13]. Quantification of the S100A11 cellular redistribution was not possible in HaCaT cells due to the small cytoplasmic volume. We therefore used human osteosarcoma (U-2 OS) cells which, in contrast to HaCaT cells, have a large cytoplasmic volume. Because U-2 OS cells express only very little endogenous S100A11 [Additional file 1: Supplemental Figure S1], we quantified the translocation event in cells expressing GFP-tagged S100A11. To simultaneously investigate whether other cellular stress stimuli are also able to induce a directed translocation of the GFP-S100A11 into the nucleus we treated the cells with extracellular Ca^2+ ^or bleomycin (BLM). Similar to endogenous S100A11 in HaCaT cells, the GFP-S100A11 construct was translocated into the nucleus of U-2 OS cells by Ca^2+ ^as well as BLM treatment (Figure 1B). Interestingly, introduction of DSBs by BLM was more effective than Ca^2+ ^in induction of S100A11 nuclear translocation as assessed in a kinetic approach. An almost complete translocation of GFP-S100A11 into the nucleus of U-2 OS cells was induced 30 minutes after BLM treatment compared to addition of Ca^2+ ^which triggered only a partial translocation to the perinuclear region at this time point. A significant translocation of S100A11 occurred only after 6 hours after Ca^2+ ^treatment. This observation suggests that the dynamics of cellular S100A11 redistribution is stress type dependent. The translocation of GFP-S100A11 was specific as cells transfected with GFP alone showed a random cellular distribution pattern of the GFP signal after induction of DNA damage by BLM [Additional file 2: Supplemental Figure S2].

**Figure 2:**
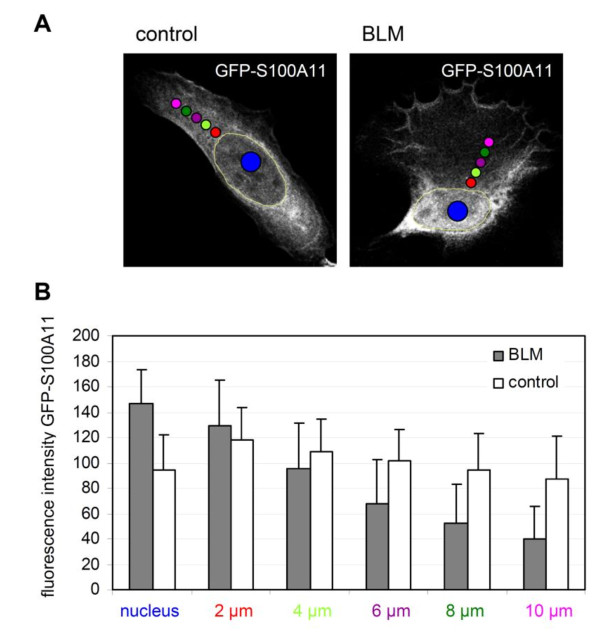
**Quantification of S100A11 translocation in stress stimulated cells**. The translocation of GFP-S100A11 in U-2 OS cells which were treated with BLM for 30 min to induce DNA double strand breaks (DSB) or in control cells was quantified by measurement of the average GFP-S100A11 fluorescence intensity in different areas within the cytoplasm as well as in the nucleus using MetaMorph analysis software as described in Materials and Methods. (A) The fluorescence intensity of GFP-S100A11 in five cytoplasmic areas (2 μm diameter each; stained in red, light green, violet, green and pink, respectively) localized at the nuclear membrane to cellular periphery in four different directions based on the nuclear membrane as well as of three areas (5 μm diameter each; stained in blue) randomly selected in the nucleus was measured radially. (B) Quantification of the GFP-S100A11 fluorescence signal in the cytoplasmatic areas and in nuclear areas of BLM-treated U-2 OS cells (n = 20) (grey column) or in control cells (n = 20) (white column). Data are displayed as mean values (±SD). Translocation of GFP-S100A11 in DNA damaged cells was significant between all examined areas.

To quantify the directed translocation of S100A11 from the cytoplasm into the nucleus we defined five circled areas (each 2 μm in diameter) localized along a line from the nuclear membrane to the cellular periphery as well as areas (5 μm diameter) randomly selected in the nucleus (Figure 2A). Altogether, a cytosolic area of at least 10 μm in distance to the nuclear membrane was analyzed by this approach. The average fluorescence intensity of the GFP-S100A11 signal of the five specific cytosolic areas was determined radially in four different cytosolic directions based on the nuclear membrane and in the nuclear areas using MetaMorph software 30 minutes after treatment with BLM (Figure 2B). With this approach we detected an increase of GFP-S100A11 signal in the nucleus of DNA damaged U-2 OS cells compared to control cells. Simultaneously, the GFP-S100A11 signal decreased in the cytoplasm of BLM-treated cells. The effect was observed in the peripheral cytosolic area. Thus, induction of DNA damage by BLM triggers a directed translocation of S100A11 from the cytoplasm into the nucleus. In contrast, the distribution of GFP-S100A11 was almost similar in various locations throughout the cytoplasm of untreated control cells (Figure 2B). Furthermore, a similar nuclear translocation of S100A11 after introduction of DSBs was also detectable in another cell line [Additional file 3: Supplemental Figure S3]. These data demonstrate that DNA damage is a powerful stress stimulus to activate a directed translocation of S100A11 into the nucleus of human cells.

### Nuclear translocation of S100A11 is mediated by nucleolin

**Figure 3:**
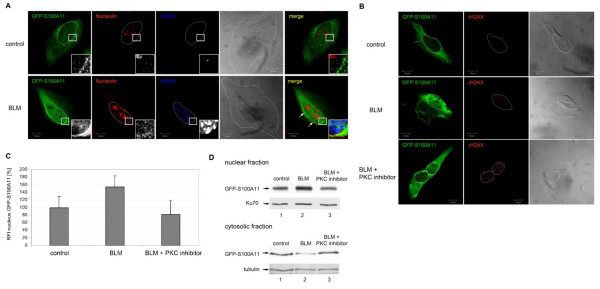
**Nucleolin distribution during nuclear translocation of S100A11 in DNA damaged cells**. (A) U-2 OS cells which expressed GFP-S100A11 were treated with BLM to induce DSB for 1 h followed by immuno-staining against GFP-S100A11 (green), nucleolin (red) and γH2AX (blue). The overlay image (merge) shows that GFP-S100A11 and nucleolin partially colocalize in the perinuclear region (labeled by arrows) in cells containing damaged DNA compared to control cells. Areas marked by a rectangle are shown enlarged in each image as insets. (B) Inhibition of phosphorylation of S100A11 by a myristolated PKC inhibitor interferes with S100A11 translocation. U-2 OS cells were transfected with a GFP-S100A11 construct and treated with BLM for 30 min or pre-treated with a PKC inhibitor for 17 hrs and subsequently with BLM. Cells were analyzed by two-color immunostaining followed by laser scanning microscopy for GFP-S100A11 (green) and γH2AX (red). (C) Quantification of the GFP-S100A11 relative fluorescence intensity (RFI) in nuclei of cells treated with BLM or BLM and a PKC inhibitor and in control cells by assessment of the GFP-S100A11 fluorescence intensity in several nuclear areas (2 μm diameter) in percent. At least 14 cells for each condition were analyzed for quantification. Data are displayed as mean values (±SD). The average of the GFP-S100A11 fluorescence intensity measured in the control approach was set as 100%. (D) U-2 OS cells which expressed GFP-S100A11 were treated with BLM or with both BLM and the myristoylated PKC inhibitor. Afterwards, cytoplasmic as well as nuclear cell extract of treated cells and, as control, untreated cells were prepared and subjected to immunoblotting using a specific antibody against S100A11. As control for equal protein loading immunoblotting against nuclear protein Ku70 or cytosolic actin using specific antibodies were carried out.

**Figure 4:**
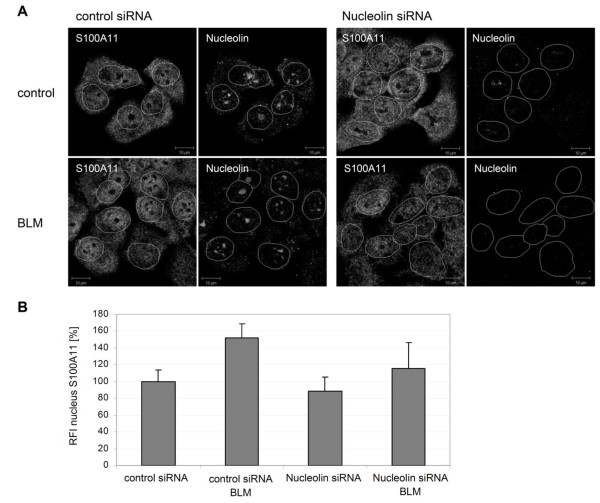
**Down-regulation of nucleolin interferes with translocation of endogenous S100A11**. (A) HaCaT cells treated with BLM for 30 min or control cells were transfected with specific siRNA for depletion of nucleolin (nucleolin siRNA) or, as a control, unspecific nonsilencing siRNA (control siRNA), respectively. Cells were analyzed by laser scanning confocal microscopy using specific antibodies against S100A11 and nucleolin. (B) Quantification of the nuclear localization of endogenous S100A11 in human HaCaT keratinocytes transfected with a specific nucleolin siRNA or, as control, an unspecific control siRNA. Transfected cells were treated with BLM for 30 min and the relative fluorescence intensity (RFI) of the S100A11 signal in selected areas (2 μm diameter) was assessed in several nuclear areas by a specific anti-S100A11 antibody as well as in untreated cells. At least 25 cells were analyzed for each condition. Data are displayed as mean values (±SD). The average of the S100A11 fluorescence intensities measured in untreated control cells transfected with control siRNA was set as 100%.

Nucleolin has been previously implicated in the cellular translocation of S100A11 after treatment with extracellular Ca^2+ ^[13]. We therefore analyzed the role of nucleolin in DNA damage-induced S100A11 nuclear accumulation. A prerequisite of the nuclear transport of S100A11 is a specific phosphorylation of S100A11 by PKCα which is activated by high Ca^2+ ^[17]. Phoshorylation of S100A11 triggers its binding to nucleolin. Nucleolin is involved in several cellular processes such as ribosomal assembly and maturation, signal transformation as well as shuttling activities [18-20]. Therefore, we assessed if nucleolin is also involved in a directed translocation of S100A11 induced by DNA damage. The induction of DSBs by BLM caused in GFP-S100A11 transfected U-2 OS cells a redistribution of a fraction of cellular nucleolin to the perinuclear region (Figure 3A). Redistribution was absent in control cells containing GFP-S100A11 without BLM treatment. A higher degree of colocalization was detected between GFP-S100A11 and nucleolin in the perinuclear region of BLM treated cells which was not detectable in controls (Figure 3A, insets). Again, an increased distribution of GFP-S100A11 in perinuclear regions and nucleus appeared after induction of DNA damage. To confirm that nucleolin is able to specifically transfer the poshosphorylated form of S100A11 into the nucleus, PKCα was inhibited using a myristoylated PKC inhibitor [21]. The cellular distribution of GFP-S100A11 and nucleolin was then examined by immunofluorescence staining followed by confocal laser scanning microscopy. Because specific phosphorylation of S100A11 is a prerequisite for nuclear transfer triggered by a high Ca^2+ ^stimulus, we expected that the phosphorylation event may be also necessary for S100A11 translocation into the nucleus induced by DNA damages. In U-2 OS cells containing damaged DNA, GFP-S100A11 was translocated directly into the nucleus again. In contrast, in DNA damaged cells incubated with the PKC inhibitor only very little GFP-S100A11 was detectable in the nucleus. GFP fluorescence intensity in these cells was similar to control cells (Figure 3B). The altered cellular distribution of GFP-S100A11 after treatment with BLM and/or the myristoylated PKC inhibitor is also reflected by the distinct GFP-S100A11 pattern in cytoplasmic as well as nuclear cell fractions (Figure 3D). To evaluate the influence of nucleolin on the nuclear translocation of endogenous S100A11 more specifically, nucleolin was depleted in HaCaT cells using siRNA (Figure 4). The amount of nuclear S100A11 was quantified in HaCaT keratinocytes under several conditions. A significant increase of nuclear S100A11 was detectable in cells with damaged DNA compared to untreated control cells. The increase of endogenous S100A11 in the nucleus is comparable to the amount of translocated GFP-S100A11 into nuclei of U-2 OS cells triggered by DNA damage. Additionally, we found similar intensities of nuclear S100A11 in nucleolin-depleted cells and in control cells. Induction of DNA DSBs by BLM in cells lacking nucleolin did result in an only slightly intensified translocation of endogenous S100A11 into the nucleus compared to untreated cells lacking nucleolin (Figure 4).

### Impeded nuclear translocation of S100A11 inhibits cell proliferation

**Figure 5:**
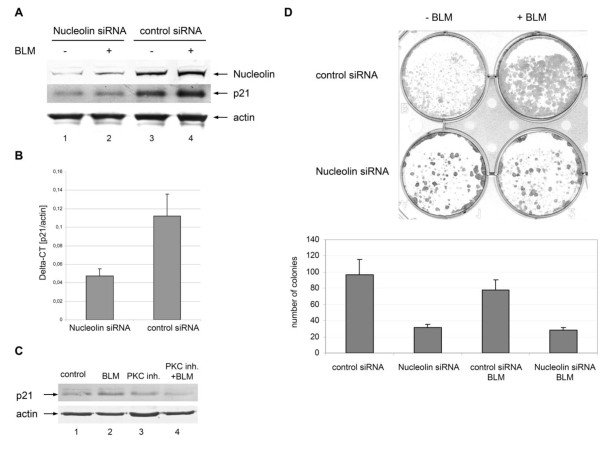
**Depletion of nucleolin induced downregulation of p21 and impeded cell proliferation**. (A) Protein extract of human HaCaT cells transfected with specific nucleolin siRNA (lanes 1-2) or unspecific control siRNA (lanes 3-4) which were treated with BLM for 30 min (lanes 2 and 4) or untreated cells (lanes 1 and 3) were subjected to immunoblotting against nucleolin and p21 using specific antibodies. As a control for equal protein loading corresponding actin levels are shown by immunoblot. (B) HaCaT keratinocytes transfected with specific nucleolin siRNA or unspecific control siRNA were cultured for 72 h and treated for 1hour with BLM (12.5 μg/ml). Then total RNA was extracted from the cells and p21 gene expression analysis was performed. The CT values of p21 were normalized to actin. Two independent experiments were performed and data are displayed as mean values (±SD). (C) HaCaT cells treated with BLM, the myristoylated PKC inhibitor, or both BLM and PKC inhibitor, and, as a control, untreated cells were lysed and protein extracts were subjected to immunoblotting using a specific antibody against p21. As a control for equal protein loading corresponding actin levels are shown by immunoblot. (D) Decreased proliferation capacity of nucleolin-depleted HaCaT cells. For assessment of the proliferation capacity of HaCaT keratinocytes, colony formation assays were carried out. HaCaT cells were transfected with a specific nucleolin siRNA or, as a control, with unspecific control siRNA, treated with BLM 72 h after transfection and grown for further 7 d (+BLM). As a control, transfected cells grown without BLM treatment (-BLM). For quantification, the colonies in 3 representative areas (1.0 cm diameter) of 6-well dishes with transfected HaCaT cells with (+BLM) or without (-BLM) BLM treatment were counted. Data are displayed as mean values (±SD).

Recently, we showed that the depletion of S100A11 leads to an inhibition of DNA damage-induced up-regulation of the cell cycle regulator p21 [5]. Therefore, we were interested whether inhibition of S100A11 translocation into nuclei after depletion of nucleolin also induces a decrease of p21. HaCaT cells transfected with specific nucleolin siRNA were treated with BLM to induce DNA DSBs for 30 min or left untreated. This analysis revealed a significant reduction of p21 protein levels in nucleolin-depleted cells (Figure 5A). The reduction of p21 was independent of BLM treatment in HaCaT cells. These observations demonstrate that nucleolin-directed translocation of S100A11 into the nucleus is required for the induction of p21 and confirm our previous results [5]. Consistent with these data, the transcriptional activation of p21 gene expression was also decreased in HaCaT cells lacking nucleolin (Figure 5B). Additionally, the p21 protein level was decreased when S100A11 phosphorylation was inhibited in cells treated with BLM (Figure 5C). Finally, we determined the proliferation capacity of nucleolin depleted HaCaT cells in colony-forming assays (Figure 5D). A significant decrease of the colony forming capacity of cells lacking nucleolin was observed (Figure 5D). Nucleolin depletion also induced an increase of the subG1/apoptotic fraction by a factor of three in FACS analyses (data not shown). Similar results were previously obtained after S100A11 knock-down [5], supporting our conclusion that nucleolin and S100A11 function in concert during DNA damage response pathways.

## Discussion

S100 proteins are involved in Ca^2+^-modulated signal transduction pathways [22,23]. A translocation of S100A11 induced by Ca^2+ ^as a cellular stress stimulus might be a key mechanism to regulate the formation of specific signalling complexes in a spatially and temporally regulated manner [13,24]. The data reported in the present study indicate a direct translocation of S100A11 into the nucleus induced by DNA damage. Recently, we showed that S100A11 seems to be involved in DNA repair processes because it functionally interacts with Rad54B which is involved in homologous recombination [5]. This interaction targets Rad54B to sites of DNA double-strand break (DSB) repair. Several members of the S100 protein family are transcriptionally regulated by factors involved in both, DNA damage repair and cell cycle control [25-27]. Additionally, it was also shown that the expression of S100A8, a further S100 family protein, was stimulated by UV-A radiation in a mouse model [28]. Directed translocation of proteins seems to be a general response to different cellular stress stimuli, including S100 family members [13,29-32]. Such nucleo/cytoplasmic translocations occur in both directions. We have shown here that nuclear translocation of S100A11 was triggered not only by Ca^2+ ^as previously described, but also by DNA damage. The accumulation of S100A11 in nuclei was more effective after DNA damage compared to the Ca^2+ ^stimulus. This may be related to very strong stress signals usually associated with DNA breaks. The S100A11 translocation triggered by DNA damage is, at least partly, mediated by nucleolin (Figure 4). Nucleolin was described as a shuttling protein which is involved in directed translocation into the nucleus of several proteins [33-35]. Interestingly, a redistribution of nucleolin from nucleoli to perinuclear regions as well as a colocalization between S100A11 and nucleolin in these regions after treatment with the DNA damaging agent BLM occurred as shown in the present study. Depletion of nucleolin by RNA interference as well as inhibition of S100A11 phosphorylation by a myristoylated PKC inhibitor inhibited the directed translocation of S100A11 into the nucleus in DNA damaged cells. Nevertheless, as minor S100A11 signal intensities still appeared in cells lacking nucleolin or cells incubated with the PKC inhibitor, it is tempting to speculate about the existence of further nuclear transfer mechanisms for S100A11. Additionally, it is conceivable that endogenous S100A11 as well as the S100A11-GFP fusion is able to diffuse passively into the nucleus due to its small molecular size.

After induction of the nuclear translocation of S100A11 by DNA damage in HaCaT keratinocytes the protein level of the cell cycle regulator p21 increased significantly. These data are consistent with recently published results showing an increased p21 protein level in DNA damaged cells [5]. The activation of p21 must be p53 independent as HaCaT cells possess only an inactive form of p53 that is not able to induce p21 [36]. Our present data indicate that S100A11 is responsible for the regulation of the p21 level in HaCaT cells. The regulation of p21 in a p53 independent manner seems to correlate with depletion of S100A11 as shown elsewhere [5] or inhibition of the S100A11 translocation into the nucleus as shown here. Our studies also revealed that depletion of nucleolin inhibits transcriptional activation of the p21 gene. This was not expected because data of Sakaguchi et al. suggested that a transcriptional activator of p21, Sp1, can be liberated from nucleolin by the binding of nuclear S100A11 to nucleolin [13]. We suggest that p21 may be controlled under these circumstances by an as yet unknown mechanism that involves S100A11. Finally, we have shown that reduced S100A11 levels in the nucleus diminished the proliferation capacity of HaCaT keratinocytes. Since p21 can act as an inhibitor of apoptosis [37] it is not surprising that the decrease of nuclear S100A11 followed by reduction of p21 induced an increased apoptotic cell fraction.

## Conclusions

On the basis of these data it seems that S100A11 is able to mediate a cellular stress stimulus induced by DNA damage by nucleolin-mediated translocation into the nucleus and regulation of p21 activity.

## Authors' contributions

TG carried the immunofluorescence-based and Western blot experiments out, generated the EGFP-S100A11 construct and was involved in analyzing the data. UM was involved in immunofluorescence analyses. TU was involved in the quantification of translocation experiments. JH carried out the qRT-PCR. PH was involved in analyzing the immunofluorescence data and helped to draft the manuscript. CM conceived the study, was involved in analyzing the data, and drafted the manuscript. All authors have read and approved the final manuscript.

## Supplementary Material

Additional file 1 **Expression analysis of S100A11 protein in different human cell lines by immunoblotting**. Protein extracts of U-2 OS osteosacroma cells (lane 1) and HaCaT keratinocytes (lane 2) were subjected to immunoblotting against endogenous S100A11 using a specific antibody. As a control for equal protein loading corresponding actin levels were shown by immunoblot. Click here for file

Additional file 2 **Distribution of GFP in DNA damaged U-2 OS cells**. U-2 OS cells were transfected with a GFP construct, treated with bleomycin (BLM; 12.5 IU/ml) for 30 min and analyzed by two-color immunostaining followed by laser scanning microscopy for GFP (green) and for γH2AX (red) 30 min after BLM treatment. Click here for file

Additional file 3 **Translocation of S100A11 into the nucleus of human A431 cells after stress stimulation**. Fixed cells treated with bleomycin (BLM) for 30 min were immunostained with anti-S100A11 antibody and anti-γH2AX antibody. In BLM treated cells increased staining of S100A11 in the nucleus can be observed. Click here for file
