# Supplementary figures and images for: DNA damage-induced translocation of S100A11 into the nucleus regulates cell proliferation
Source: BMC Cell Biol. 2010 Dec 17;11:100. doi: 10.1186/1471-2121-11-100 (PMC3018407; doi:10.1186/1471-2121-11-100)

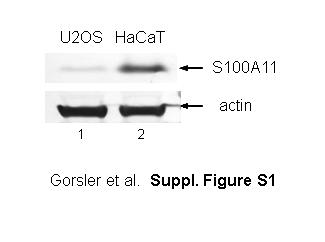

Supplement: Additional file 1 — Expression analysis of S100A11 protein in different human cell lines by immunoblotting. Protein extracts of U-2 OS osteosacroma cells (lane 1) and HaCaT keratinocytes (lane 2) were subjected to immunoblotting against endogenous S100A11 using a specific antibody. As a control for equal protein loading corresponding actin levels were shown by immunoblot. [file 1471-2121-11-100-S1.TIFF]

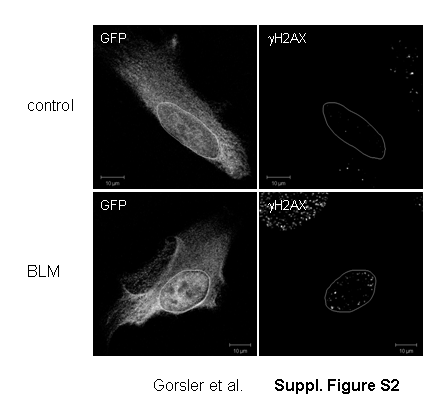

Supplement: Additional file 2 — Distribution of GFP in DNA damaged U-2 OS cells. U-2 OS cells were transfected with a GFP construct, treated with bleomycin (BLM; 12.5 IU/ml) for 30 min and analyzed by two-color immunostaining followed by laser scanning microscopy for GFP (green) and for γH2AX (red) 30 min after BLM treatment. [file 1471-2121-11-100-S2.TIFF]

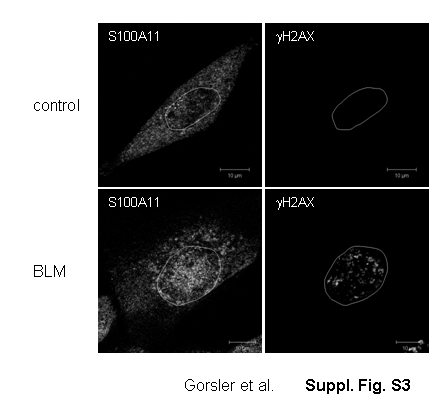

Supplement: Additional file 3 — Translocation of S100A11 into the nucleus of human A431 cells after stress stimulation. Fixed cells treated with bleomycin (BLM) for 30 min were immunostained with anti-S100A11 antibody and anti-γH2AX antibody. In BLM treated cells increased staining of S100A11 in the nucleus can be observed. [file 1471-2121-11-100-S3.TIFF]
